# The effectiveness of virtual reality exercise games on balance functions and fear of falling in women with osteoporosis

**DOI:** 10.1007/s00296-024-05569-6

**Published:** 2024-03-22

**Authors:** Nihal Yilmaz, Meryem Kösehasanoğulları

**Affiliations:** 1 Department of Physical Medicine and Rehabilitation, Uşak University Medical School, Uşak, Turkey; 2 Department of Physical Medicine and Rehabilitation, Adana Cıty Training and Research Hospital, Adana, Turkey

**Keywords:** Exercises, Exergames, Balance, Osteoporosis

## Abstract

To investigate and compare the effectiveness of Nintendo Wii games and home exercises on balance functions in patients with osteoporosis, an important disease adversely affecting balance functions. The patients included in the study were randomized into two groups the Wii exercise group (*n* = 30) and the home exercise group (*n* = 30). Wii exercise group performed balance exercises with a Nintendo Wii device and balance board three times a week for 12 weeks under the supervision of a physiotherapist in the hospital, and home exercise group was prescribed home exercises three days a week for 12 weeks. Balance functions were evaluated with the timed up-and-go-test and Berg Balance Scale, and the fall risk was evaluated with the Falls Efficacy Scale at the beginning and end of 12 weeks of treatment. Comparison of pre- and post-treatment timed up-and-go-test, Berg Balance Scale, and Falls Efficacy Scale results in both groups revealed statistically significant improvements (*p* = 0.001; *p* < 0.05). Furthermore, post-treatment test scores between the two groups demonstrated a significant enhancement in Wii exercise group regarding the Berg Balance Scale score (Mean ± SD 52.9 ± 3.63) (*p* = 0.001; *p* < 0.05). Within the osteoporotic population, balance functions serve as robust predictors of fall risk. Improvement in balance functions is crucial for the prevention of falls and subsequent osteoporotic fractures. In our study, we found that balance exercises performed with Wii games are effective in improving balance functions in patients with osteoporosis.

## Introduction

According to the definition provided by the World Health Organization (WHO), osteoporosis is characterized as a progressive and systemic skeletal disorder marked by a reduction in bone mass, deterioration in bone microarchitecture, or the occurrence of fragility fractures [1]. In elderly individuals with osteoporosis, the risk of falls escalates due to diminished standing ability, compromised dynamic balance, and reduced walking speed. 45% of individuals over the age of 65 experience at least one fall per year [2]. Falls are a significant risk factor in the development of osteoporotic fractures [3]. The occurrence of fractures associated with osteoporosis may lead to diminished functional capacity, elevated morbidity and mortality rates, and escalating expenses in healthcare [4].

In addition to pharmacological interventions, exercise therapy is recommended for the prevention and treatment of osteoporosis and related fractures. Systematic reviews and meta-analyses have demonstrated the positive impact of exercise modalities, as non-pharmacological approaches, on bone density in postmenopausal women. Exercises play a crucial role in reducing bone density loss by maintaining both cortical and trabecular bone density [5]. Specifically, balance exercises, by stimulating proprioceptive receptors and enhancing reaction times, are pivotal in preventing falls [3, 6, 7]. Physical exercise proves to be a cost-effective approach, providing favourable effects on fall risk and bone strength without significant adverse effects [5].

Advancements in technology have led to the increased availability of video games, which are now employed for therapeutic purposes. Virtual reality-based programs or exergaming, including video game consoles like Nintendo Wii and Microsoft Xbox, are among the applications used to promote physical activity [8]. Exergaming has demonstrated positive effects on balance functions in various conditions such as stroke, multiple sclerosis (MS), Parkinson's disease, cerebral palsy, and older adults [9].

Despite osteoporosis significantly impacting balance functions and the documented positive effects of home exercises, there is limited literature exploring the effectiveness of exergaming in osteoporosis patients [10]. Consequently, this study aims to comparatively investigate the effectiveness of home exercises and exergaming with Wii games in improving balance functions in individuals with osteoporosis.

## Methods

### Sample size calculation

In this study, the sample size was calculated with an alpha error value of 0.05, a power of 80%, and an effect size of 0.75. Following a power analysis, it was determined that a minimum of 27 patients were required for each group.

### İnclusion criteria

The gold standard in the diagnostics of osteoporosis is the dual-energy X-ray absorptiometry (DXA) examination [11]. According to the WHO criteria BMD measurements, the patients with an L1–L4 T-score of <  − 2.5 and femoral neck T-score of <  − 2.5 were considered to have osteoporosis [12]. Patients diagnosed with osteoporosis according to WHO criteria and who agreed to participate in the study were included after obtaining informed consent.

### Exclusion criteria

Patients with neurological diseases, vertigo, cognitive impairment, and vision and hearing problems that could cause balance disorder as well as those who regularly exercised over the past six months were excluded from the study.

This research constitutes a randomized controlled trial. Informed consent was obtained from all participating patients. Initially comprising 68 patients, the study excluded 7 individuals who did not meet criteria (2 for vertigo, 2 for cerebrovascular events, 2 for regular exercise, and 1 for hearing loss). An additional patient declined participation due to transportation difficulties. Consequently, the study enrolled a total of 60 female patients aged over 45 years, diagnosed with osteoporosis, between March 1 and September 1, 2022, from the Physical Therapy and Rehabilitation Department. The average duration of osteoporosis in the patients was 11.66 ± 8.17 years. All patients were receiving appropriate pharmacological treatment for osteoporosis. There were no patients with osteoporotic fractures.The participants were randomly assigned to two groups, the Wii exercise group (WEG) and the home exercise group (HEG).

In WEG, supervised by a physiotherapist in the hospital, participants performed Nintendo Wii-based balance exercises three times a week for 12 weeks. HEG, on the other hand, was advised to undertake home exercises three days a week for the same duration. Participants in both groups were instructed to perform exercises as eight repetitions in two sets twice a day and received weekly follow-up calls to assess adherence.

Before commencing exercises, both groups engaged in a five-minute brisk walk as a warm-up. Strengthening, aerobic, and balance exercises for upper and lower extremities, as well as the trunk, were conducted in group settings as eight repetitions in two sets. Subsequently, participants played a selected balance board game from the Wii screen to enhance balance. The VR program utilized the Wii Fit game, employing a Wii balance board and Wii remote control. This program focused on weight transfer, support, and balance improvement. Games simulating activities like climbing stairs aimed at enhancing aerobic capacity, muscle endurance, and strength [13].

The exercise session for patients not experiencing palpitations, shortness of breath, blackout, or dizziness was completed within 45 min. HEG participants received joint range of motion exercises for the neck, upper extremities, trunk, and lower extremities, in addition to stretching, isometric strengthening, and balance exercises, as demonstrated by the physiotherapist. Exercise sessions for both groups were terminated if participants reported dizziness, blackout, palpitations, or a sensation of falling during the exercises [13].

### Outcome measures

#### Timed up and go (TUG)

The TUG test involved patients starting in a seated position in a chair and then getting up to walk a predetermined three-meter flat surface at a normal pace. Subsequently, they returned to the chair, and the elapsed time for the entire process was recorded [14].

#### Berg Balance Scale (BBS)

The BBS is a clinical test designed to assess balance and fall risk in older adults [15]. Comprising 14 items, it evaluates performance based on the ability to execute balance tasks, with scores ranging from 0 (unable to perform the task) to 4 (able to perform the task independently and safely). The highest achievable score is 56, with scores categorized as 0–20 indicating a balance disorder, 21–40 indicating acceptable balance, and 41–56 indicating good balance. The validity and reliability analyses of the Turkish version of BBS were undertaken by Şahin et al. [16].

#### Falls Efficacy Scale (FES)

Developed by Tinetti et al., the FES aims to evaluate an individual's concern about falling during daily activities [17]. The scale consists of 10 items, each scored from 1 (very confident) to 10 (not confident at all). A total score exceeding 70 points suggests a notable fear of falling in the individual. The Turkish validity andreliability of the Fall Efficiency scale was performed by Ulus et al. [18].

### Statistical analysis

The statistical analyses were conducted using SPSS v.24.0. Descriptive statistics were applied to evaluate the study data, and the data distribution was assessed using the Kolmogorov–Smirnov test. The Mann–Whitney *U* Test was employed for comparing quantitative data between groups with non-normally distributed data. The Friedman test was utilized to compare pre-and post-treatment measurement results within the study groups (Table 2). Significance was considered at the *p* < 0.05 levels. Spearman correlation tests were applied for correlation analyses. The coefficients used in the study are defined according to the Pearson method.

## Results

**Fig. 1:**
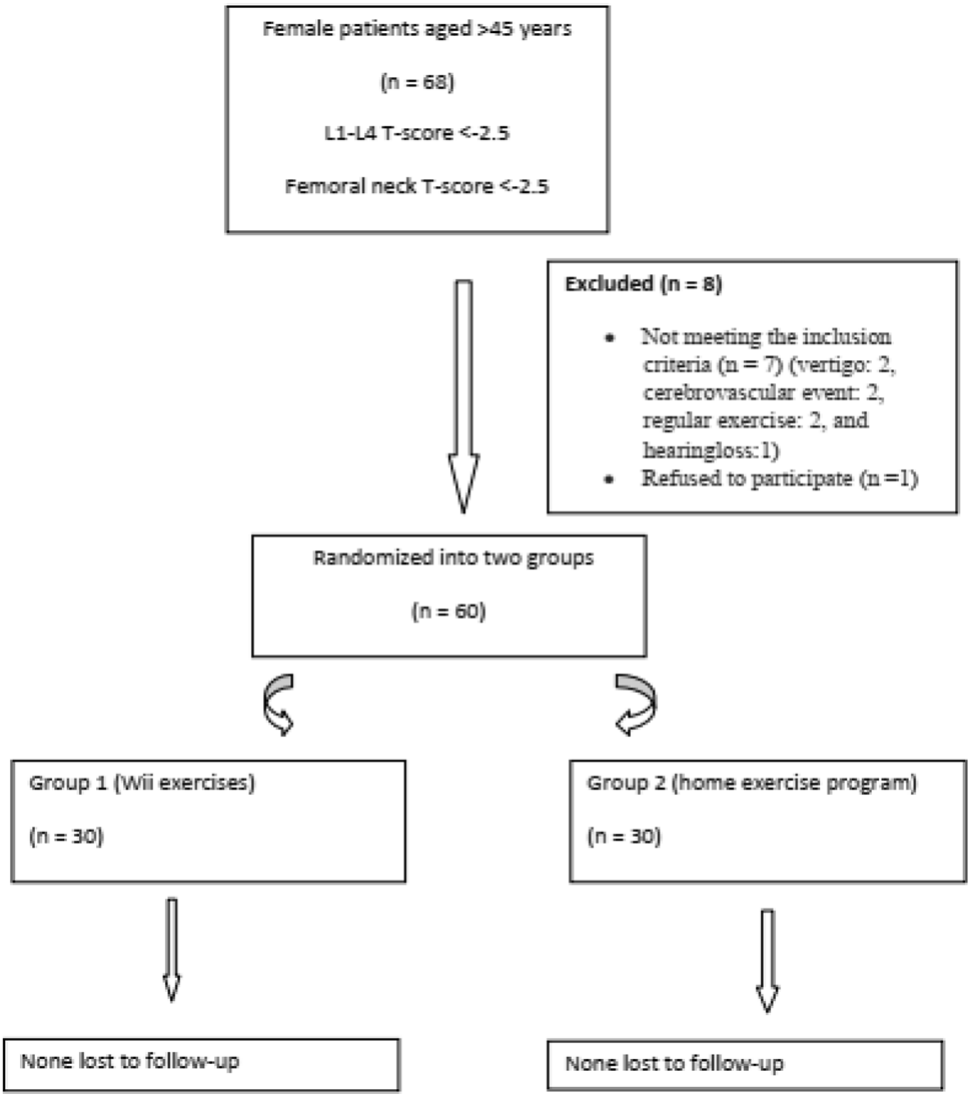
Study flow chart

Among the 68 patients initially assessed for eligibility, seven did not meet the inclusion criteria, and one declined participation, resulting in a sample of 60 patients (Fig. 1).

The mean age of patients was 67 (± 10.64) years in the Wii Exercise Group (WEG) and 68 (± 9.06) years in the Home Exercise Group (HEG). Statistically significant improvements were observed in both groups when comparing TUG (8.78 ± 1.36) (*p* = 0.001; *p* < 0.05), BBS (52.9 ± 3.63) (*p* = 0.001; *p* < 0.05), and FES (33.77 ± 11.77) (*p* = 0.001; *p* < 0.05) scores before and after treatment (Table 1).

**Table 1:** Comparison of the pre- and post-treatment measurement results of the study groups

	Pre-treatment	Post-treatment	*p*
WEG			
BBS			
Mean ± SD	42.7 ± 3.15	52.9 ± 3.63	
Min–max	34–48 (43)	44–56 (54.5)	0.001**
(median)			
TUG			
Mean ± SD	10.81 ± 1.79	8.78 ± 1.36	
Min–max	6.89–14 (11.3)	7–11.79 (8.55)	0.001**
(median)			
FES			
Mean ± SD	43.67 ± 14.09	33.77 ± 11.77	
Min–max	26–78 (39)	19–56 (34)	0.001**
(median)			
HEG			
BBS			
Mean ± SD	41.93 ± 2.26	47.1 ± 2.89	
Min–max	38–46 (42)	42–52 (47)	0.001**
(median)			
TUG			
Mean ± SD	11.09 ± 1.55	9.59 ± 1.75	
Min–max	7.9–13.5 (11.1)	7.2–13.6 (9.8)	0.001**
(median)			
FES			
Mean ± SD	37.2 ± 16.6	29.67 ± 14.54	
Min–max	13–70 (36.5)	12–57 (26.5)	0.001**
(median)			

*SD* standard deviation, *WEG* Wii exercise group, *HEG* home exercise group, BBS Berg Balance Scale, *TUG* timed up and gotest, *FES* Fall efficacy test

Friedman test ^**^p < 0.01. Statistically significant improvements were observed in both groups when comparing TUG, BBS, FES

Comparing post-treatment scores between the two groups, a statistically significant enhancement in the Berg Balance Scale (BBS) test was observed in WEG (Mean ± SD 52.9 ± 3.63) (*p* = 0.001; *p* < 0.05). However, no statistically significant difference was found concerning Timed Up and Go (TUG) (8.78 ± 1.36) (*p* = 0.072) (*p* > 0.05) and Falls Efficacy Scale (FES) (33.77 ± 11.77)(*p* = 0.133) (*p* > 0.05) scores (Table 2).

**Table 2:** Comparison of the post-treatment measurements of the study groups

	*n*	Mean ± SD	Min–max (median)	*p*
Berg balance scale				
WEG	30	52.9 ± 3.63	44–56 (54.5)	0.001**
HEG	30	47.1 ± 2.89	42–52 (47)	
Timed up and go test				
WEG	30	8.78 ± 1.36	7–11.79 (8.55)	0.072
HEG	30	9.59 ± 1.75	7.2–13.6 (9.8)	
Falls efficacy scale				
WEG	30	33.77 ± 11.77	19–56 (34)	0.133
HEG	30	29.67 ± 14.54	12–57 (26.5)	

*SD* standard deviation, *WEG* Wii exercise group, *HEG* home exercise group

Mann–Whitney *U* test

^*^p < 0.05. Comparing post-treatment scores between the two groups, a statistically significant enhancement in the Berg Balance Scale (BBS) test was observed in WEG

^**^p < 0.01

The inter-group comparison of the difference between pre-treatment and post-treatment BBS scores revealed statistical significance favouring WEG (Mean ± SD 10.2 ± 2.85) (*p* = 0.001; *p* < 0.05). However, no statistically significant result was detected in the comparison of the difference between pre-treatment and post-treatment TUG scores (2.03 ± 1.15)(0.095) (*p* > 0.05) (Table 3).

**Table 3:** Inter-group evaluation of the differences between the pre- and post-treatment BBS and TUG scores

	*n*	Mean ± SD	Min–max (median)	*p*
BBS score difference				
WEG	30	10.2 ± 2.85	4–15 (10)	0.001**
HEG	30	5.17 ± 2.34	− 1 to 10 (6)	
TUG score difference				
WEG	30	− 2.03 ± 1.15	− 4.1 to 0.19 (-2.05)	0.095
HEG	30	− 1.5 ± 1.02	− 3.3 to 1.1 (− 1.5)	

*BBS* Berg Balance Scale, *TUG* timed up and go test, *SD* standard deviation, *WEG* Wii exercise group, *HEG* home exercise group, *FES* Fall Efficacy Scale

Mann–Whitney *U* test

^*^p < 0.05. The inter-group comparison of the difference between pre-treatment and post-treatment BBS scores revealed statistical significance favouring WEG

^**^p < 0.01

Examining the correlation between age and TUG and FES scores of WEG before and after treatment, no statistically significant results were found. Regarding the correlation between age and BBS of WEG, a negative and moderately significant relationship was identified between age and both pre-treatment scores of BBS (*r* =  − 0.468, *p* < 0.01) and post-treatment scores of BBS (*r* =  − 0.410, *p* < 0.05).

For HEG, a positive and weakly significant relationship was found between age and TUG post-treatment scores (*r* = 0.394, *p* < 0.05). No correlation was found between age and TUG pre-treatment scores, and there was no statistically significant correlation between FES and BBS pre-treatment and post-treatment scores (*p* > 0.05).

## Discussion

Balance disorders leading to falls and fractures are prevalent in individuals with osteoporosis [19]. Effective management of static balance is crucial in reducing the risk of falls in older adults [20]. Proprioceptive rehabilitation emphasizes the importance of balance exercises to prevent falls [6]. Exergaming, a recent intervention in balance rehabilitation, has shown promise in improving balance in various neurological conditions, including multiple sclerosis (MS), stroke, Parkinson’s disease, and cerebral palsy. Given the positive effects observed in diverse diseases, this study aimed to explore the effectiveness of exergaming in enhancing balance among patients with osteoporosis. Statistical comparison revealed a significantly greater improvement in the WEG compared to the HEG, consistent with existing literature (*p* < 0.05). Furthermore, both groups exhibited statistically significant improvements in post-treatment Berg Balance Scale (BBS), Timed Up and Go (TUG), and Falls Efficacy Scale (FES) scores compared to their pre-treatment values.

Previous research supports the positive impact of balance exercises, including dual-task balance training, performed with a balance device in osteoporosis patients [21]. A review by Cho et al. highlighted the efficacy of Nintendo Wii exercises in reducing falls, alleviating related concerns, and enhancing balance and lower extremity muscle strength in the elderly [22]. Consistent with these findings, our study underscores the effectiveness of exergaming in improving balance among osteoporosis patients. A study by Gilani et al. in 2023 similarly demonstrated the effectiveness of virtual reality exergaming in enhancing the balance functions of osteoporosis patients and reducing the risk of falling [23]. Kim SH et al., in a study resembling ours, reported positive effects of balance exercises on balance functions and fear of falling in an elderly population, albeit not specifically osteoporotic and isolated individuals [20].

In a systematic review, Calaifore et al. analyzed seven randomized controlled trials from 2013 to 2020 involving 209 patients with MS. Among these trials, 97 patients underwent exergaming treatment for balance improvement, demonstrating a significant enhancement in balance test results, particularly in Berg Balance Scale (BBS) scores [24]. A meta-analysis by Chan et al. in 2021, encompassing 23 randomized controlled studies investigating the effects of exergaming therapy on balance in stroke patients, revealed significant benefits in favour of exergaming, particularly when utilizing Nintendo Wii [25].

In summary, our study supports the effectiveness of exercise gaming in improving balance in patients with osteoporosis. It is consistent with the current literature and highlights its potential as an effective rehabilitation tool.

### Limitations

One notable limitation of this study is the ownership of the Nintendo Wii device by the researchers. The inability to provide each patient with a device for home use necessitated that WEG conducted their exercises under supervision in the hospital. This lack of access to the device for home-based exercises introduces a potential bias in the exercise setting, as patients in the HEG were unable to benefit from one-on-one supervision during their exercises. Although efforts were made to assess exercise compliance in the HEG through weekly calls, the absence of direct supervision could have impacted compliance negatively, serving as another limitation of the study.

Furthermore, the study's design as a single-centre trial is another limitation. The single-centre approach may restrict the generalizability of the findings to a broader population, and variations in healthcare settings or patient demographics could influence the external validity of the results. Future research with a multi-centre design could enhance the generalizability of findings and provide a more comprehensive understanding of the intervention's effectiveness across diverse populations and settings.

## Conclusion

Building upon the insights gained from our research, a notable finding is the superior efficacy of exercises performed with the Nintendo Wii compared to traditional home exercises. This aligns with prior studies which highlighted the positive impact of exergaming on reducing falls and improving balance in the elderly and osteoporosis patients, respectively. Our results reinforce the growing body of evidence supporting the benefits of incorporating exergames, particularly those utilizing Wii technology, into osteoporosis management strategies.
